# Engineered adeno-associated virus 3 vector with reduced reactivity to serum antibodies

**DOI:** 10.1038/s41598-021-88614-9

**Published:** 2021-04-29

**Authors:** Mika Ito, Naomi Takino, Takamasa Nomura, Akihiko Kan, Shin-ichi Muramatsu

**Affiliations:** 1 Division of Neurological Gene Therapy, Center for Open Innovation, Jichi Medical University, Tochigi, Japan; 2 KAINOS Laboratories, Inc., Tokyo, Japan; 3 Center for Gene and Cell Therapy, The Institute of Medical Science, The University of Tokyo, Tokyo, Japan

**Keywords:** Biological techniques, Biotechnology, Medical research

## Abstract

The natural serotypes of adeno-associated virus (AAV) or their variants, such as AAV8 and AAV5, are commonly used as vectors in the clinical programs for liver-targeted gene therapy. While AAV8 vectors are not highly efficient at targeting primary human hepatocytes, AAV3 vectors have recently demonstrated remarkable efficiency at targeting both human and non-human primate hepatocytes. However, the presence of high levels of neutralizing antibodies (NAbs) impedes transduction into hepatocytes, representing a major obstacle to the clinical application of AAV3 vectors. Herein, we engineered the viral capsid to reduce its reactivity with pre-existing NAbs, thereby enhancing the transduction efficiency. By introducing three substitutions (S472A, S587A, and N706A) on the surface loop of AAV3B capsid protein, we generated a triple mutant AAV3 (AAV.GT5) vector with less reactivity to anti-AAV capsid NAbs. While the transduction efficiency of AAV.GT5 into human hepatocellular cell lines was similar to those of parental AAV3B, it was 50-fold higher for hepatocytes derived from humanized mice compared to AAV8 vectors. Moreover, the AAV.GT5 vector yield was similar to those of the AAV2 and AAV3B vectors. Thus, high resistance to pre-existing NAbs makes AAV.GT5 a promising candidate for future liver-targeted gene therapy clinical trials.

## Introduction

Adeno-associated virus (AAV) vector-mediated gene delivery has demonstrated promising therapeutic effects in many clinical trials. Currently three AAV gene therapy products, authorized by the US Food and Drug Administration and the European Medicine Agency, are available: Glybera (alipogene tiparvovec) for lipoprotein lipase deficiency, Luxtruna (voretigene neparvovec-rzyl) for inherited retinal degeneration, and Zolgensma (onasemnogene abeparvovec-xioi) for spinal muscular atrophy^1^. These products employ AAV1, AAV2, and AAV9, respectively, as vectors^1^. Moreover, AAV vectors have been successfully used in liver-targeted gene therapy, most notably for hemophilia^2–5^. However, AAV-based liver-directed gene therapies must overcome several challenges prior to their clinical applications^6^. A major hurdle is the poor efficacy associated with transduction into human hepatocytes as compared to that into mouse liver cells, caused by species-specific differences in AAV tropism between mice and non-human primates. Although AAV8-based vectors are approximately 10- to 100-fold more efficient than AAV2- or AAV5-based vectors in mouse liver transduction, accumulating preclinical and clinical data indicate that AAV8- and AAV5-based vectors, which are used in most current clinical protocols are not efficient at specifically targeting primary human hepatocytes. Further, the data from preclinical models are not predictive of their clinical performance in humans^7,8^.

Another hurdle facing liver-directed gene therapy is the high prevalence of neutralizing antibodies (NAbs), which diminishes the efficacy of AAV-based therapies after systemic administration. Even low titers, such as 1:17 for AAV2^9^ and 1:1 for AAV-Spark100 (engineered capsid derived from AAVrh74)^3^, have been associated with reduced, or abrogated, therapeutic efficacy. Thus, the presence of NAbs against AAV capsids is a common exclusion criterion for participation in the clinical trials. The seroprevalence rates against wild type AAV in humans vary depending on various factors such as age, geographic location, and species of origin of the capsid, and can reach 30% for AAV5, 40% for AAV8, and 70% for AAV1 and AAV2^10^. This substantially reduces the proportion of the population that could benefit from AAV- based therapeutics. Due to the high degree of conservation in the amino acid sequence among AAVs, cross-reactivity of anti-AAV antibodies between a wide range of serotypes is typically > 50%. Therefore, a substantially large proportion of the population cannot benefit from AAV-based therapeutics unless the vectors are designed to evade NAbs.

It is, therefore, imperative to explore effective approaches for the enhancement of AAV transduction and simultaneous NAbs evasion. Recently, studies have shown that AAV3 vectors were able to efficiently transduce human hepatocytes in a humanized mouse model^8,11^ and non-human primate^7^. Moreover, the novel synthetic liver-trophic capsid AAV-LK03 generated by a directed evolutional approach shares > 90% capsid homology with AAV3^12^. Another liver-trophic vector, AAV3B-DE5, was recently reported by applying directed evolution specifically to AAV3^13^. In this study, we employed a bioengineering process to rationally design the AAV3 capsid. Triple substitution of amino acids, S472A, S587A, N706A, on the surface loop of the AAV3B capsid protein was studied to assess their transduction efficiency into primary human hepatocytes and enhanced resistance to pre-existing human NAbs.

## Results

### Bioengineering of AAV3

**Figure 1:**
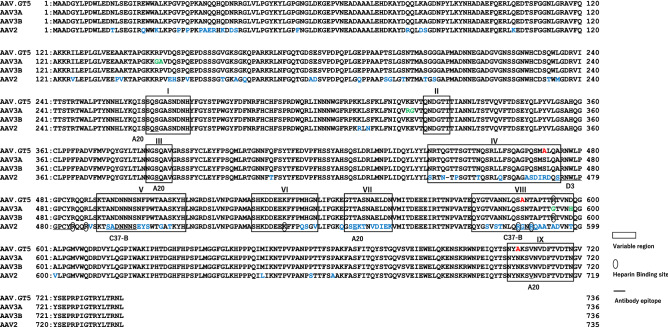
Comparison of VP1 amino acids of AAV.GT5 with AAV2, AAV3A, and AAV3B. Surface variable regions (VR-1 to VR-IX) are boxed. Amino acid residues that bind to heparan sulfate are indicated with ellipse. Epitopes of antibodies against AAV2 (A20, D3, and C37-B) are underlined. Substitution of three amino acids, S472A, S587A, and N706A, on the surface loop of AAV3 are shown in red. Amino acids in AAV2 and AAV3A that are different from those in AAV3B are shown in blue and green, respectively.

Based on the previous reports on the capsid structures of AAV2, AAV3A, AAV3B, AAV8, AAVrh8, and AAV9^14–16^, as well as antigenic epitope mapping of AAV1, 2, 5 and AAV8^17^, we identified three variable regions (IV, VIII, and IX) in the surface loops of VP3 as important for reducing the immunogenicity. We substituted three amino acids, one in each variable region considered to be involved in antibody binding, with alanine (S472A, S587A, N706A; Fig. 1). These substitutions did not affect the yield of the vector. We routinely obtained 5 × 10^3^ vg/cell of AAV.GT5. The percentage of full particle was 82% for AAV2, 80% for AAV3B, 69% for AAV8, 54% for AAV-Spark100, 59% for AAVhu37, and 74% for AAV.GT5. The yield and percentage of full particles of AAV.GT5 were comparable to those of AAV3B vectors.

### Efficient transduction into human hepatocytes by AAV.GT5

**Figure 2:**
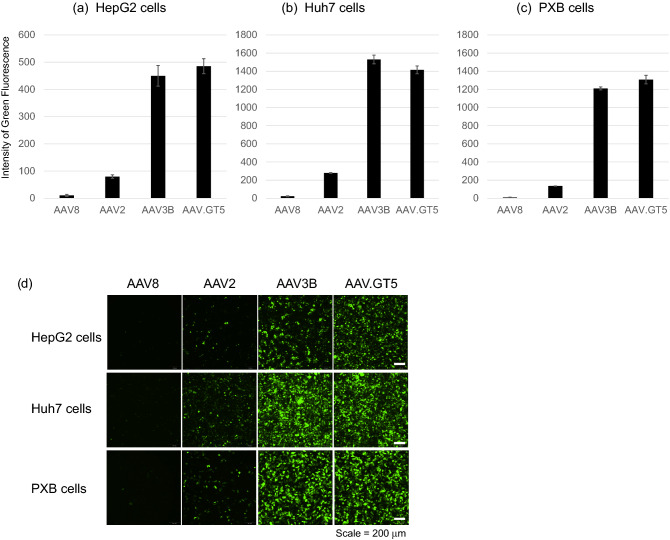
Transduction efficiency of AAV8, AAV2, AV3B, and AAV.GT5 on human hepatocytes. Seven days after the infection of green fluorescence protein (GFP)-expressing AAV vectors at the 10^4^ of multiplicity of infection, mean fluorescence intensity (MFI) of GFP were measured. (**a**) HepG2 cells, (**b**) Huh7 cells, (**c**) PXB cells. The scale of the vertical axis is different for each cell-line. (**d**) Representative images. Scale bar represents 200 μm.

We compared the transduction efficiency of AAV.GT5 with those of AAV8, AAV2, and AAV3B in two human hepatocellular carcinoma cell lines, HepG2 and Huh7 cells, as well as PXB cells, which are human hepatocytes derived from chimeric mice with human liver^18^. Seven days after the infection with green fluorescence protein (GFP)-expressing AAV vectors at a 10^4^ multiplicity of infection (MOI) in HepG2 cells, the mean fluorescence intensity (MFI) of GFP (n = 4) were found to be 10.8 for AAV8, 79.8 for AAV2, 449.8 for AAV3B, and 485.3 for AAV.GT5. Normalization of the MFI with respect to that of AAV8 showed that the GFP expression levels caused by AAV2, AAV3B, and AAV.GT5 were 7, 42, and 45 times greater than that by AAV8, respectively (Fig. 2a). Similarly, in Huh7 cells, the GFP levels (n = 4) were 12, 68, and 63 times greater in cells infected with AAV2, AAV3B, and AAV.GT5 than those infected by AAV8, respectively (Fig. 2b). In PXB cells, the transduction efficiencies of AAV2, AAV3B, and AAV.GT5, as indicated by GFP levels (n = 5), were 15, 135, and 145 times greater than that of AAV8, respectively (Fig. 2c). Representative images are shown in Fig. 2d.

**Figure 3:**
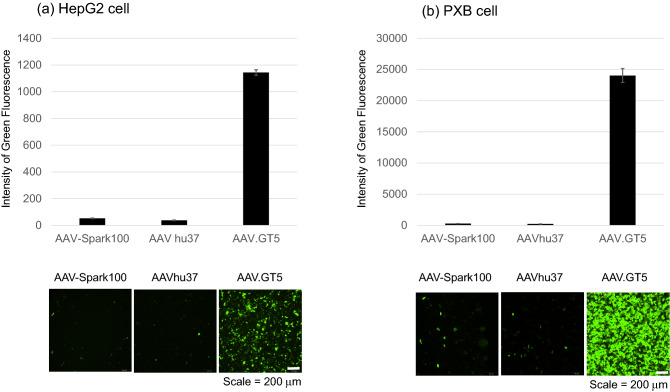
Transduction efficiency of AAV.GT5, AAV-Spark100, and AAVhu37 on human hepatocytes. Nine days after the infection of green fluorescence protein (GFP)-expressing AAV vectors at the 4 × 10^4^ of multiplicity of infection, mean fluorescence intensity (MFI) of GFP were measured. The scale of the vertical axis is different for each cell-line. (**a**) HepG2 cells, (**b**) PXB cells. Representative images are shown in the lower panels. Scale bar represents 200 μm.

We then compared the transduction efficiency of AAV.GT5 with clinically applied vectors, AAV-Spark100 or AAVhu37. These vectors are members of the hepatotropic Clade E family and share 93.9% and 93.5% amino acid identity with AAV8, respectively^19^. HepG2 and PXB cells were infected with each of the three AAV vectors individually. Nine days after infection, the MFI caused by the GFP expression was measured and compared. Following AAV vector infection at 5 × 10^4^ MOI, HepG2 cells showed MFI of 52.7 for AAV-Spark100, 38.0 for AAVhu37, and 1144.0 for AAV.GT5 (Fig. 3). In PXB cells, the infection with each AAV vector at 5 × 10^4^ MOI led to MFI of 280.5 for AAV-Spark100, 230.5 for AAVhu37, and 24,025.8 for AAV.GT5 (Fig. 3).

**Figure 4:**
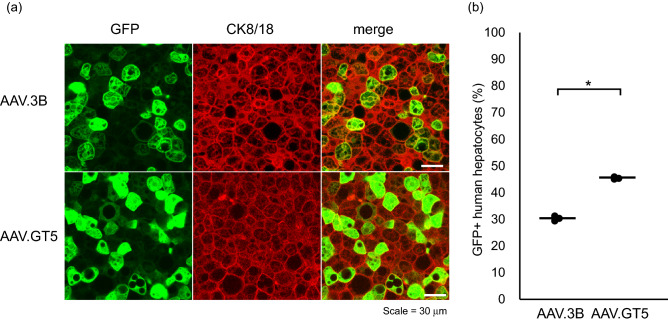
(**a**) Visualization of AAV vectors transduction of human hepatocytes in a chimeric mouse liver. Representative images of hepatocytes in PXB mice that received 1.0 × 10^11^ vg/mouse of AAV3B or AAV.GT5 expressing GFP. Green stain, GFP; red stain, human hepatocytes (CK8/18 +). Scale bar represents 30 μm. (**b**) Comparison of transduction efficacies of AAV3B and AAV.GT5 by quantifying the percent GFP + human hepatocytes in liver sections. The black circles represent the efficiency for each mouse, and the bars represent the average. *P = 0.00001.

Efficient transduction of human hepatocytes by AAV.GT5 was also demonstrated in the liver of chimeric PXB mice^18^. Intravenously administrated 1.0 × 10^11^ vg/mouse of AAV3B or AAV.GT5 resulted in GFP expression in 30% or 45% in human hepatocytes, respectively (Fig. 4). These efficiencies are comparable with those in previous studies using AAV3B and its similar capsids in various human xenograft mouse models^8,11,13^. In these mice, more than 85% of mice hepatocytes were replaced by human hepatocytes, while most of the remaining mice hepatocytes were unhealthy due to the associated genetic background. Thus, we could not assess the transduction efficiency of AAV.GT5 on mice hepatocytes.

### Lower seroprevalence and cross-reactivity of antibodies against AAV.GT5

**Figure 5:**
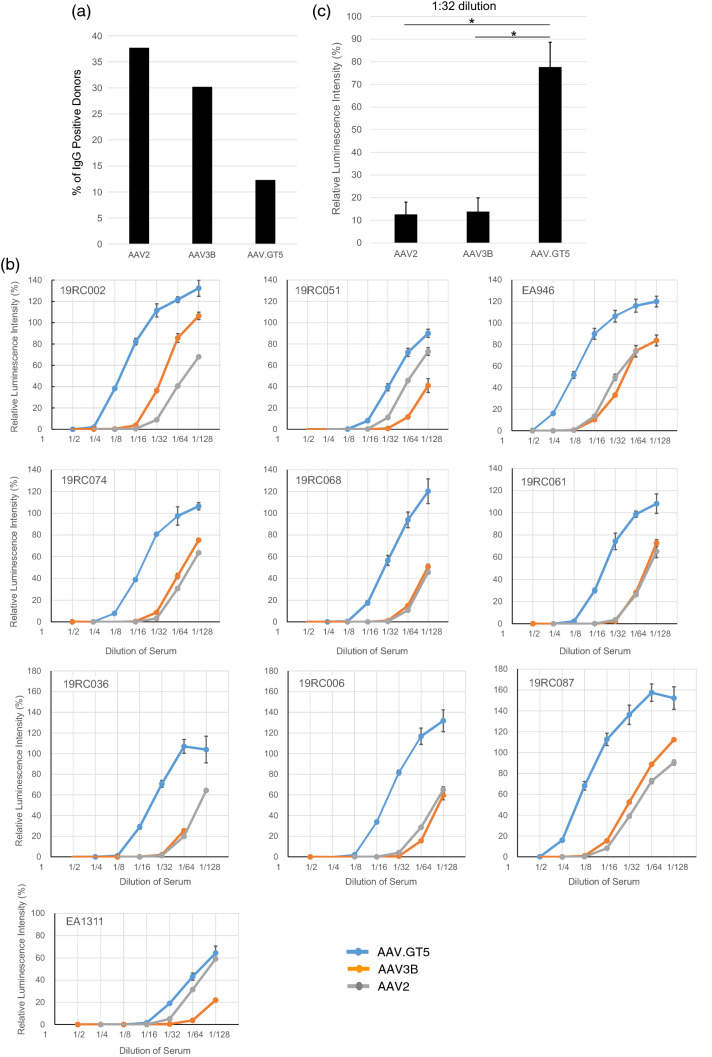
(**a**) Prevalence of IgG against AAV capsids in healthy human subjects. The percentage of total capsid-specific IgG is shown for each AAV vectors for 106 healthy adults. (**b**) Comparison of inhibitory effects of NAbs on transduction efficiency. In ten serum samples that reduced transduction of AAV2 vectors by more than 50% at 1:32 dilution, transduction was inhibited at higher concentrations in AAV.GT5 vectors than in AAV3B vectors, suggesting these sera had lower cross-reactivity against AAV.GT5 vectors. (**c**) Mean inhibitory effects of NAbs in ten serum samples on transduction efficiency. At 1:32 dilution of the sera, the expression of luciferase by the AAV2 vector was reduced to 12.6% of that without the serum. The expression of luciferase by the AAV3B vector was also reduced to 13.8%, while that with AAV.GT5 vector remained 77.7%. *P = 0.00001.

**Figure 6:**
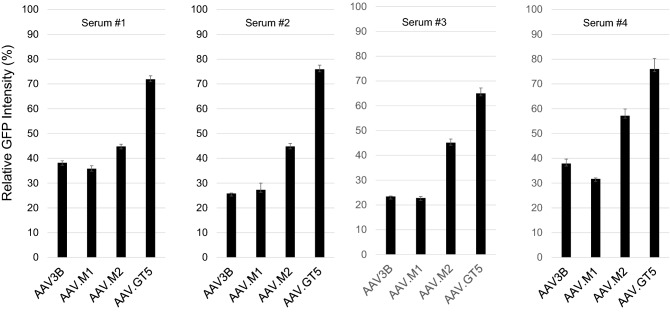
Comparison of NAb inhibitory effects on transduction efficiency. In four serum samples that reduced transduction of AAV2 vectors by more than 50% at 1:32 dilution, transduction was less inhibited in AAV.GT5 vectors than in AAV3B, AAV.M1, or AAV.M2 vectors, suggesting these sera had lowest cross-reactivity against AAV.GT5 vectors. On the vertical axis, the intensity of GFP in the control, which was not mixed with serum containing the NAbs, was set to 100%. Relative GFP intensities were measured in four wells for each vector.

We measured the levels of immunoglobulin G (IgG) antibodies against AAV.GT5, AAV2, and AAV3B in 106 healthy adults. To capture AAV-specific IgG antibodies in an enzyme-linked immunosorbent assay (ELISA), each vector particle was immobilized on plates. NAb titer 1:32 corresponded to an optical density (OD) of 0.527 for AAV.GT5, 0.349 for AAV2, and 0.486 for AAV3B in the ELISA (Supplementary Table S1). When these values were used as cutoff points, seroprevalence of IgG against AAV2, AAV3B and AAV.GT5 was found to be 37.7%, 30.2%, and 12.3%, respectively (Fig. 5a, Supplementary Table S2). All 13 sera that were IgG positive against AAV.GT5 were also found to be cross-reactive with AAV2 and AAV3B. We compared the inhibitory effects of NAbs against AAV.GT5, AAV2, and AAV3B on transduction efficiency in ten serum samples (Supplementary Table S3) that showed a reduction in transduction of AAV2 by more than 50% at 1:32 or higher dilution (NAb titer 1:32). In these sera, the transduction of AAV.GT5 was more efficient than that of AAV3B (Fig. 5b,c). We also compared the inhibitory effects of NAbs against AAV.GT5, AAV.M1 (S587A), AAV.M2 (S472A and S587A), and AAV3B on transduction efficiency in four serum samples. Inhibitory effects were weakest in AAV.GT5 compared to single or dual mutants (Fig. 6). We then measured the avidity of IgG antibodies using the ELISA with chaotropic agents in five sera (NAb titer 1:32). Avidity index was highest in AAV.GT5 followed by AAV3B and AAV2 (Supplementary Table S4).

## Discussion

The liver is a preferred target for gene therapy against several genetic and metabolic disorders^20^, because it synthesizes proteins and detoxifies various metabolites. To date, most clinical gene therapies directed to the liver using AAV vectors have focused on hemophilia. However, initial gene therapy trial for hemophilia B using the AAV2 vector, a prototype of the AAV vector, has failed to show significant clinical benefits, although AAV2 vectors had previously demonstrated efficient transduction into mouse hepatocytes^9^. This led to the realization that there are several obstacles to overcome in this field. (1) Results from preclinical studies on animals do not necessarily translate directly into humans. Species or even strain differences in the transduction efficiency of AAV vectors have led to disappointing results in the development of vectors. For example, engineered capsid AAV.PHB.B, which exhibited broad transduction in the brain of some mouse strains, was found to not be so effective in other strains of mice and non-human primates^21^. (2) The high prevalence of NAbs to AAV capsids hinders systemic administration of the vectors^10,22–24^. Although successful liver transduction was reported with AAV5 vector in the presence of pre-existing anti-AAV5 NAb titers up to 1:1,030 for non-human primates and 1:340 for humans^25^, it remains to be elucidated if this evasion from NAbs is specific for AAV5 vectors. (3) Transgene expression declines following elimination of transduced hepatocytes^26^. (4) High doses of the vectors are more likely to elicit a host immune response. Possible dose-dependent toxicity of AAV vectors has recently been highlighted by the deaths of three patients in a clinical trial for the X-linked myotubular dystrophy in which 3 × 10^14^ vg/kg of AAV8 vectors was systemically administered^27,28^. Therefore, it is imperative to explore effective approaches for the enhancement of AAV transduction and NAb evasion.

In this study, we engineered AAV3. There are two isolates of wild type AAV3, AAV3A^14^ and AAV3B^15^. These isolates differ in eight amino acids of capsid proteins, although functional differences between the two isolates have not been elucidated. We selected AAV3B as the parent AAV for capsid modification, since most of the previous reports on AAV3 used AAV3B. AAV3 uses the human hepatocyte growth factor (HGF) receptor as a co-receptor for cellular entry^29^ and has been shown to efficiently transduce human hepatocytes^7,8,11,30^. Recent structural analysis by X-ray crystallography and cryo-electron microscopy on various AAVs suggested that the surface topology of capsid proteins are conserved across all AAVs^16,31^. Nine common variable regions (VR-I to VR-IX) were identified among AAV2, AAV8, AAVrh8, and AAV9^16^. Differences in the surface loops of these VRs affect receptor binding, transduction efficiency, and antigenicity. Possible epitopes of antibodies against AAV capsids have been mapped using various approaches including peptide scanning, peptide insertion, and site-directed mutagenesis^17^. For example, epitopes of AAV2 and the footprint of C37-B antibody include residues 492–498 on VR-V and 585–589 on VR-VIII^31^, while peptide scanning using D3 antibody identified residues 474–483 on VR-IV^32^, and site-directed mutagenesis mapped multiple residues including 263, 264, 384, 385, 548, and 708 for A20 antibody^33^. Since the capsid VP1 proteins from AAV2 and AAV3B were 83% identical^15^, we predicted that NAbs that cross-react with AAV2 and AAV3B also recognize these residues.

Previous mutagenesis studies on the AAV2 capsid protein demonstrated negative effects after specific amino acid substitutions. With E563A and H526A substitutions, infectivity was severely reduced^33,34^. E563A substitution also eliminated capsid protease activity^35^. With Y704A substitution, second-strand synthesis and transcription were impaired^36^. It is also important not to interfere with the function of assembly activating protein (AAP), which is translated across the start codon of VP3^37^. Although the binding site of HGF on the AAV3 capsid has not been elucidated, residue 594 has been identified as a binding site for heparan sulfate proteoglycan (HSPG)^38^.

Based on these findings, we selected residues 472, 587, and 706 from VR-IV, VR-VIII, and VR-IX, respectively, for engineering the AAV3 capsid. These three residues are conserved between AAV3A and AAV3B, however are diverse in other AAVs. We used a conventional strategy by substituting the amino acids in these position with alanine, a small amino acid, to eliminate effective interactions between capsid proteins and NAbs. Although rational design is still challenging and substitution to alanine may enhance the antibody binding^39^, we confirmed that prevalence of AAV-specific IgG and NAb activity were reduced in AAV.GT5 with S472A, S587A, and N706A substitutions. Since addition of chaotropic agents such as guanidine thiocyanate or urea did not reduce the avidity of the AAV-specific IgG in AAV.GT5 compared with AAV3B, triple mutations may affect the accessibility of epitopes rather than the binding strength of the NAbs. Moreover, we observed efficient transduction of AAV.GT5 into primary human hepatocytes derived from chimeric mice with human liver (PXB cells), as well as into two human hepatocellular carcinoma cell lines. The transduction efficiency of AAV.GT5 was comparable with that of parental AAV3B, and significantly greater than that of AAV8, AAVrh37, and AAV-Spark100. Although PXB cells recapitulate many aspects of human hepatocytes, it remains unclear how well the human xenograft model could predict transduction efficiency in the human body^40^. As an approach to address this concern, we have recently initiated in vivo transduction studies on non-human primates comparing AAV.GT5 expressing human coagulation factor IX with other liver-directed AAV vectors.

In the ELISA method used in this study, the AAV vector particles immobilized on plates captured AAV-specific antibodies. Using whole vector particles as antigens without degradation, antibodies that are specific to each AAV, including NAbs, can be assessed^41^. In the wild type AAV seropositive individuals, IgG1 appears to be the predominant immunoglobulin subclass, and titers of IgG1 correlate well with the level of NAbs^22^. As we set up cutoff points corresponding to 1:32 NAb titers, prevalence of IgG against AAV2 and AAV3B was lower than that reported in most previous studies where lower cutoff values were used; however, it is difficult to compare the results from different laboratories using non-standardized assays with variable sensitivities^10,22–24^. We also showed that the prevalence of IgG and titers of NAbs against AAV.GT5 were lower than those against AAV3B. Still, there were a substantial number of people who had low NAb titers against AAV.GT5. However, the lower antibody titers against AAV.GT5 can be mitigated by strategies such as evasion of pre-existing NAbs, as demonstrated on AAV5 in a hemophilia study^24^, or treatment with protease that degrades circulating IgG^42,43^, to harness the advantages of AAV.GT5 over other AAVs for clinical applications. Furthermore, high transduction efficiency and low reactivity toward NAbs may allow administration of relatively low doses, which could have potential benefits in terms of reduced immunogenicity and also allow for the inclusion of more number of patients in need of gene therapy.

In summary, we bioengineered AAV3 for efficient transduction of the vector into primary human hepatocytes and to alleviate the reactivity with the pre-existing NAbs without affecting its preparation yields.

## Materials and methods

### Vectors

To design AAV3 mutants based on the findings of previous AAV studies, we chose amino acid residues based on the following criteria^14–17^: (1) located on the surface loop of variable regions; (2) located in or around the epitope of ant-AAV2 antibodies; (3) conserved between AAV3A and AAV3B; (4) less likely to affect capsid formation or genome incorporation; (5) expected to enhance transduction of human hepatocytes^33^. AAV vector plasmids contained an expression cassette consisting of a human cytomegalovirus immediate-early (CMV) promoter followed by cDNA encoding GFP or luciferase and a simian virus 40 polyadenylation signal sequence. The expression cassette was located between the inverted terminal repeats of the AAV3A genome. The AAV.GT5 *vp* cDNA was synthesized by introducing three amino acid substitutions (S472A, S587A, and N706A) in AAV3B *vp.* For comparison, a single amino acid mutant AAV.M1 (S587A), and double amino acid mutants AAV.M2 (S472A and S587A) *vp* cDNAs were synthesized. Recombinant AAV vectors were produced by transient transfection of human embryonic kidney (HEK293) cells, as previously described^44^. The cells were maintained in Dulbecco’s Modified Eagle’s Medium and Harn’s F-12 Nutrient Mixture (DMEM/F12, Thermo Fisher Scientific, Waltham. MA) supplemented with 10% heat-inactivated fetal bovine serum (FBS, Sigma-Aldrich, St. Louis, MO), 1% penicillin and streptomycin (PenStrep, Thermo Fisher Scientific). The cells were transfected with the vector plasmid, the AAV3B *rep* and either AAV2 (Gene Bank Accession NC_001401.2), AAV3B (AF028705.1), AAV.GT5, AAV8 (NC_006261), AAV-Spark100^3^, or AAVhu37 (AY530600.1) *vp* expression plasmids, and the adenoviral helper plasmid pHelper (Agilent Technologies). The recombinant viruses were purified by isolation from two sequential continuous CsCl gradients. Viral titers were determined by qPCR. Empty-to-full particle ratios were determined by direct counting of the electron micrographs (Hanaichi Ultrastructure Research Institute. Okazaki, Japan). Empty particles were distinguished based on the electron-dense centers following negative staining with uranyl acetate.

### In vitro transduction

Human hepatocellular carcinoma HepG2 and Huh7 cells were purchased from the Japanese Collection of Research Bioresources Cell Bank (Tsukuba, Japan). Primary human hepatocytes derived from chimeric mice with human liver (PXB mice) were obtained from PhoenixBio Co., Ltd. (Higashi-Hiroshima, Japan)^18^. HepG2 and Huh7 cells were maintained in DMEM low glucose (Thermo Fisher Scientific) with 10% FBS and 1% PenStrep. PXB cells were cultured in dHCGM (PhoenixBio Co., Ltd.) consisting of DMEM with 10% FBS, 20 mM HEPES, 44 mM NaHCO_3_, 1% PenStrep, 15 μg/mL l-proline, 0.25 μg/mL insulin, 50 nM dexamethasone, 5 ng/mL epidermal growth factor, 0.1 mM l-ascorbic acid, and 2% dimethyl sulfoxide.

HepG2 or Huh7 cells were plated at a density of 5 × 10^4^ cells per well in a 96-well optical bottom plates (Thermo Fisher Scientific). Twenty-four hours after seeding, HepG2 or Huh7 cells were infected with 5 × 10^8^ vg/well of GFP expressing AAV2, AAV3B, AAV.GT5, and AAV8. PXB cells were plated at a density of 7 × 10^4^ cells per well in a 96-well plate. Six days after seeding, PXB cells were infected with 5 × 10^8^ vg/well of GFP expressing AAV2, AAV3B, AAV.GT5, and AAV8. For comparison of AAV.GT5, AAV-Spark100, and AAVhu37, 2 × 10^9^ vg/well for HepG2 cells and 3.5 × 10^9^ vg/well for PXB cells were infected. Seven to nine days after infection, GFP expression was measured using a plate reader (BioTech Japan, Tokyo, Japan).

### In vivo transduction

Mouse studies were approved by the Animal Care and Use Committee at Jichi Medical University (17203-02). All the methods were carried out in compliance with the relevant guidelines and regulations including the ARRIVE guidelines. PXB mice contained a transgene containing an albumin promoter/enhancer and urokinase-type plasminogen activator cDNA in a severe combined immunodeficient background (cDNA-uPA^wild/+^/SCID)^18^. Human hepatocytes were transplanted via the spleens. Only mice with serum human albumin levels were > 7.0 mg/mL (corresponding to > 70% humanized) were used for transduction experiments. AAV3B or AAV.GT5 vectors expressing the GFP transgene were diluted in 200 mL of phosphate-buffered saline (PBS) and injected into the 20-week-old male PXB mice through the tail vein at 1.0 × 10^11^ vg/mouse (n = 3 for each cohort).

### Immunohistochemistry

At day 14 after vector injection, mice were perfused with 0.01 M PBS under deep anesthesia, followed by 4% paraformaldehyde. The livers were removed and cut into three blocks. The tissue blocks were rinsed for 3 days in PBS containing 30% sucrose. The blocks were cut on a freezing microtome into 40 μm-thick sections. The sections were then incubated with primary antibodies against GFP (diluted 1:500; Abcam Inc., Hudson, WI), or human-specific CK8/18 (diluted 1:50; PROGEN, Heidelberg, Germany) in PBS containing 0.3% Triton X-100 at 4 °C for 24 h. They were then incubated with Alexa Fluor 488 goat anti-chicken IgY (diluted 1:500; Thermo Fisher Scientific, Waltham, MA) and Alexa Fluor 594 goat anti-mouse IgG (diluted 1:500; Thermo Fisher Scientific) for 1 h at 24 °C. Immunoreactivity was assessed and viewed under a microscope (BZ-9000; Keyence, Japan) and using a confocal laser scanning microscope (FV10i; Olympus, Tokyo, Japan). The numbers of GFP and CK8/18 double-immunoreactive cells were counted in liver sections. For sections obtained from each of the three blocks, six fields of view at 72 × were counted. A total of 1254–1702 CK8/18-immunoreactive cells in three sections were sampled per animal for cell counting.

#### Immunological assays

 Serum samples were surplus of blood donors for the Japan Red Cross Society (Tokyo). The samples were donated by an application for the use of blood donated in Japan based on the “Guidelines on the use of donated blood in R&D, etc.” Informed consent was obtained from all participants. All experiments were performed in accordance with these guidelines and approved by the institutional ethical committee of KAINOS Laboratories, Inc. (study number 2019-1). Details of the gender and age of the blood donors were not disclosed, however, they were healthy adults over 20 years of age and included both sexes. For the NAbs assay, heat-inactivated sera were continuously diluted two-fold with FBS. AAV2, AAV3B, and AAV.GT5 vectors that express luciferase under the CMV promoter were diluted with buffer containing 50 mmol/L HEPES and 150 mmol/L NaCl (pH 7.4) up to 1.6 × 10^6^ vector genome (vg)/mL. Five microliters of diluted serum were added to 5 μL diluted AAV vectors, and the mixture was incubated for 1 h at 37 °C. The mixture was then added to 96-well plates containing 2 × 10^4^ HEK293 cells. One day after transduction, luciferase expression was measured with a luciferase assay kit (Promega, Madison, WI, USA). The NAb titer was determined as the serum dilution at which 50% of AAV reporter gene expression is permitted, compared to no serum controls. For the comparison of inhibitory effects of NAbs against AAV.GT5, AAV.M1 (S587A), AAV.M2 (S472A and S587A), and AAV3B on transduction efficiency, GFP expression vectors were incubated with the serum containing NAbs against AAV2 (titer 1:32).

AAV-specific IgG were detected using an ELISA as previously described^41^. Ninety-six-well microtiter plates (Thermo Fisher Scientific) were coated with either 70 ng of AAV2, 500 ng of AAV3B, or 70 ng of AAV.GT5 vector particles per well. After blocking with 2% bovine serum albumin (BSA) in PBS, the plates were washed with 2% sucrose. Serum samples diluted at 1:1,000 with PBS/1% BSA were added to each well (100 μL/well). The plates were incubated for 1 h at 25 °C and washed three times with PBS/0.05% Tween 20. A horseradish peroxidase-conjugated anti-human immunoglobulin G (HRP-IgG) was prepared using *N*-(6-Maleimidocaproyloxy)sulfosuccinimide, sodium salt (sulfo-EMCS, DOJINDO Laboratories, Kumamoto, Japan) as a maleimide compound. A solution containing 0.1 μg/mL HRP-IgG was added to each well at a final concentration of 75 μg/mL for AAV2, 70 μg/mL for AAV3B, or 25 μg/mL for AAV.GT5. The plates were incubated for 1 h at 25 °C and washed three times with washing buffer. The color was developed by adding 100 μL 3,3′,5,5′-tetramethylbenzidine/urea hydrogen peroxide (Neogen, Lexington, KY, USA) and incubating the plates for 30 min at 20 °C. The color development was stopped by adding 1 mol/L sulfuric acid (100 μL/well), and the OD was measured at 450/640 nm.

To examine the antibody avidity in five serum samples containing NAbs (titer > 1:32) against AAV2, AAV3B and AAV.GT5, guanidine thiocyanate or urea was added to each well at final concentrations of 0.9 M and 5.0 M, respectively before HRP-IgG was added to the ELISA plates.

### Statistical analysis

Statistical analysis was performed using *R* software. Analysis of variance (ANOVA) was performed to determine differences for comparison among multiple experimental conditions. The Bonferroni post hoc test was used when comparing the conditions. Results are presented as mean ± SEM. Statistical significance was set at *P* < 0.05.

## Supplementary information

Supplementary Information.

## Data Availability

All data generated or analyzed during this study are included in this published article and its Supplementary Information files.
