# Supplementary material for: Engineered adeno-associated virus 3 vector with reduced reactivity to serum antibodies
Source: Sci Rep. 2021 Apr 29;11:9322. doi: 10.1038/s41598-021-88614-9 (PMC8084969; doi:10.1038/s41598-021-88614-9)
Supplement: Supplementary file 1 — Supplementary Information. [file 41598_2021_88614_MOESM1_ESM.docx]

***Supplementary Information***

**Engineered adeno-associated virus 3 vector with reduced reactivity to serum antibodies**

**Mika Ito^1^, Naomi Takino^1^, Takamasa Nomura^2^, Akihiko Kan^2^, and Shin-ichi Muramatsu^1,3,^***

^1^Division of Neurological Gene Therapy, Center for Open Innovation, Jichi Medical University, Tochigi, Japan

^2^KAINOS Laboratories, Inc., Tokyo, Japan

^3^Center for Gene & Cell Therapy, The Institute of Medical Science, The University of Tokyo, Tokyo, Japan

*Correspondence should be addressed to S.M. (muramats@jichi.ac.jp)

**Supplementary Table S1.** Optical densities of control samples.

| Control | AAV.GT5 | | | AAV2 | | | AAV3B | | |
| --- | --- | --- | --- | --- | --- | --- | --- | --- | --- |
|  | Sample  ID | OD | Nab  titer | Sample  ID | OD | Nab titer | Sample  ID | OD | Nab titer |
| Negative | EA-1307 | 0.030 | < 1:1 | EA-947 | 0.094 | < 1:16 | EA-942 | 0.087 | 1:1 |
| Positive | 2002H | 0.527 | 1:64 | 19RC036 | 0.349 | 1:64 | 19RC006 | 0.486 | 1:64 |

For positive controls, samples with an antibody titer of 1:32 were prepared by diluting samples with a titer of 1:64.

**Supplementary Table S2.** Immunoglobulin G antibody levels against AAV2, AAV3B, and AAV.GT5.

| **Sample** | **AAV2** | **AAV3B** | **AAV.GT5** |  | **Sample** | **AAV2** | **AAV3B** | **AAV.GT5** |
| --- | --- | --- | --- | --- | --- | --- | --- | --- |
| **1** | 0.312 | 0.134 | 0.038 |  | **28** | 0.192 | 0.103 | 0.030 |
| **2** | 0.114 | 0.081 | 0.031 |  | **29** | 0.830 | 1.023 | 0.363 |
| **3** | 0.247 | 0.805 | 0.295 |  | **30** | 0.250 | 0.121 | 0.131 |
| **4** | 0.193 | 0.431 | 0.225 |  | **31** | 0.091 | 0.086 | 0.033 |
| **5** | 0.315 | 0.305 | 0.110 |  | **32** | 0.221 | 0.163 | 0.050 |
| **6** | 0.319 | 0.477 | 0.057 |  | **33** | 0.116 | 0.155 | 0.058 |
| **7** | 0.998 | 1.214 | 0.413 |  | **34** | 0.303 | 0.154 | 0.092 |
| **8** | 0.314 | 0.534 | 0.188 |  | **35** | 1.201 | 1.729 | 0.654 |
| **9** | 1.170 | 1.410 | 0.505 |  | **36** | 0.200 | 0.089 | 0.032 |
| **10** | 0.303 | 0.147 | 0.079 |  | **37** | 0.941 | 1.155 | 0.421 |
| **11** | 0.494 | 0.255 | 0.140 |  | **38** | 0.312 | 0.280 | 0.088 |
| **12** | 0.201 | 0.090 | 0.034 |  | **39** | 0.398 | 0.232 | 0.071 |
| **13** | 0.165 | 0.163 | 0.063 |  | **40** | 0.580 | 1.130 | 0.067 |
| **14** | 0.188 | 0.080 | 0.032 |  | **41** | 0.498 | 2.489 | 0.850 |
| **15** | 0.158 | 0.095 | 0.041 |  | **42** | 2.131 | 2.309 | 0.464 |
| **16** | 0.561 | 0.370 | 0.116 |  | **43** | 0.319 | 0.130 | 0.053 |
| **17** | 0.227 | 0.181 | 0.023 |  | **44** | 0.513 | 0.764 | 0.307 |
| **18** | 0.241 | 0.150 | 0.071 |  | **45** | 0.307 | 0.583 | 0.193 |
| **19** | 0.820 | 1.234 | 0.451 |  | **46** | 1.139 | 0.162 | 0.060 |
| **20** | 0.142 | 0.098 | 0.031 |  | **47** | 1.176 | 1.251 | 0.449 |
| **21** | 1.627 | 2.209 | 0.836 |  | **48** | 0.110 | 0.089 | 0.027 |
| **22** | 0.319 | 0.152 | 0.046 |  | **49** | 0.417 | 0.655 | 0.077 |
| **23** | 0.247 | 0.123 | 0.063 |  | **50** | 0.149 | 0.113 | 0.036 |
| **24** | 0.397 | 0.249 | 0.334 |  | **51** | 0.772 | 1.409 | 0.493 |
| **25** | 0.161 | 0.109 | 0.039 |  | **52** | 0.113 | 0.096 | 0.034 |
| **26** | 0.478 | 0.620 | 0.240 |  | **53** | 0.087 | 0.080 | 0.029 |
| **27** | 0.508 | 0.174 | 0.032 |  | **54** | 0.178 | 0.122 | 0.041 |

**Supplementary Table S2 (continued)**

| **Sample** | **AAV2** | **AAV3B** | **AAV.GT5** |  | **Sample** | **AAV2** | **AAV3B** | **AAV.GT5** |
| --- | --- | --- | --- | --- | --- | --- | --- | --- |
| **55** | 1.512 | 2.467 | 0.971 |  | **81** | 0.201 | 0.202 | 0.121 |
| **56** | 0.264 | 0.192 | 0.069 |  | **82** | 1.359 | 1.494 | 0.562 |
| **57** | 0.231 | 0.084 | 0.026 |  | **83** | 0.431 | 0.349 | 0.115 |
| **58** | 0.162 | 0.091 | 0.029 |  | **84** | 0.196 | 0.104 | 0.039 |
| **59** | 0.186 | 0.067 | 0.023 |  | **85** | 0.446 | 0.195 | 0.076 |
| **60** | 0.307 | 0.102 | 0.033 |  | **86** | 0.161 | 0.105 | 0.044 |
| **61** | 0.172 | 0.160 | 0.051 |  | **87** | 3.839 | 4.0＜ | 2.911 |
| **62** | 0.172 | 0.102 | 0.052 |  | **88** | 0.266 | 0.438 | 0.271 |
| **63** | 0.109 | 0.083 | 0.057 |  | **89** | 0.169 | 0.075 | 0.025 |
| **64** | 0.209 | 0.084 | 0.040 |  | **90** | 0.326 | 0.234 | 0.088 |
| **65** | 3.315 | 4.0＜ | 1.990 |  | **91** | 0.827 | 0.956 | 0.441 |
| **66** | 0.184 | 0.356 | 0.127 |  | **92** | 0.156 | 0.212 | 0.033 |
| **67** | 0.153 | 0.110 | 0.065 |  | **93** | 1.488 | 2.410 | 0.991 |
| **68** | 0.279 | 0.133 | 0.040 |  | **94** | 3.072 | 3.410 | 1.567 |
| **69** | 0.158 | 0.130 | 0.043 |  | **95** | 0.493 | 0.188 | 0.055 |
| **70** | 0.443 | 0.188 | 0.045 |  | **96** | 0.158 | 0.111 | 0.047 |
| **71** | 0.217 | 0.141 | 0.077 |  | **97** | 0.177 | 0.075 | 0.045 |
| **72** | 0.158 | 0.099 | 0.043 |  | **98** | 0.146 | 0.077 | 0.035 |
| **73** | 0.349 | 0.228 | 0.094 |  | **99** | 0.124 | 0.081 | 0.038 |
| **74** | 0.140 | 0.105 | 0.084 |  | **100** | 3.253 | 3.918 | 2.063 |
| **75** | 0.625 | 1.827 | 0.642 |  | **101** | 0.439 | 0.504 | 0.218 |
| **76** | 0.893 | 1.314 | 0.495 |  | **102** | 0.287 | 0.350 | 0.031 |
| **77** | 0.842 | 1.692 | 0.623 |  | **103** | 0.156 | 0.189 | 0.086 |
| **78** | 1.346 | 1.782 | 0.651 |  | **104** | 0.121 | 0.131 | 0.060 |
| **79** | 0.210 | 0.107 | 0.071 |  | **105** | 0.168 | 0.100 | 0.063 |
| **80** | 1.352 | 1.515 | 0.517 |  | **106** | 0.351 | 0.095 | 0.036 |

Positive sera are highlighted in red text.

**Supplementary Table S3.** Neutralizing antibody titers against AAV.GT5, AAV3B, and AAV2 in 10 serum samples.

| Sample ID | AAV.GT5 | AAV3B | AAV2 |
| --- | --- | --- | --- |
| 19RC002 | 1:8 | 1:32 | 1:64 |
| 19RC051 | 1:32 | 1:128 ≦ | 1:64 |
| EA-946 | 1:4 | 1:32 | 1:32 |
| 19RC006 | 1:16 | 1:64 | 1:64 |
| 19RC036 | 1:16 | 1:64 ≦ | 1:64 |
| 19RC061 | 1:16 | 1:64 | 1:64 |
| 19RC074 | 1:16 | 1:64 | 1:64 |
| 19RC068 | 1:16 | 1:64 | 1:128 ≦ |
| 19RC087 | 1:8 | 1:32 | 1:64 |
| EA-1311 | 1:128 | 1:256 ≦ | 1:128 |

**Supplementary Table S4.** Avidity of IgG antibodies detected by ELISA with chaotropic agents in five serum samples (NAb titer > 1:32).

Sample ID: 20RA035

| Additive  Vector | No  Additive | 0.9M Guanidine thiocyanate | 5M Urea |
| --- | --- | --- | --- |
| AAV2  Avidity Index (%) | 1.105  100 | 0.721  **65** | 1.022  **93** |
| AAV3B  Avidity Index (%) | 1.853  100 | 1.271  **69** | 1.535  **83** |
| AAV.GT5  Avidity Index (%) | 0.552  100 | 0.470  **85** | 0.520  **94** |

Sample ID: 20RA044

| Additive  Vector | No  Additive | 0.9M Guanidine thiocyanate | 5M Urea |
| --- | --- | --- | --- |
| AAV2  Avidity Index (%) | 0.477  100 | 0.326  **68** | 0.421  **88** |
| AAV3B  Avidity Index (%) | 0.816  100 | 0.633  **78** | 0.691  **85** |
| AAV.GT5  Avidity Index (%) | 0.257  100 | 0.227  **88** | 0.240  **93** |

Sample ID: 20RA076

| Additive  Vector | No  Additive | 0.9M Guanidine thiocyanate | 5M Urea |
| --- | --- | --- | --- |
| AAV2  Avidity Index (%) | 0.881  100 | 0.577  **66** | 0.786  **89** |
| AAV3B  Avidity Index (%) | 1.462  100 | 1.080  **74** | 1.239  **85** |
| AAV.GT5  Avidity Index (%) | 0.470  100 | 0.400  **85** | 0.434  **92** |

**Supplementary Table S4 (continued)**

Sample ID: 20RA041

| Additive  Vector | No  Additive | 0.9M Guanidine thiocyanate |
| --- | --- | --- |
| AAV2  Avidity Index (%) | 0.450  100 | 0.178  **40** |
| AAV3B  Avidity Index (%) | 2.327  100 | 1.531  **66** |
| AAV.GT5  Avidity Index (%) | 0.700  100 | 0.439  **63** |

Sample ID: 20RA075

| Additive  Vector | No  Additive | 0.9M Guanidine thiocyanate |
| --- | --- | --- |
| AAV2  Avidity Index (%) | 0.562  100 | 0.298  **53** |
| AAV3B  Avidity Index (%) | 1.707  100 | 1.288  **76** |
| AAV.GT5  Avidity Index (%) | 0.527  100 | 0.412  **78** |

Avidity Index = optical density (OD) with additive/OD without additive × 100
